# Systematic Review of Health Disparities for Cardiovascular Diseases and Associated Factors among American Indian and Alaska Native Populations

**DOI:** 10.1371/journal.pone.0080973

**Published:** 2014-01-15

**Authors:** Rebecca Newlin Hutchinson, Sonya Shin

**Affiliations:** 1 Lankenau Medical Center, Wynnewood, Pennsylvania, United States of America; 2 Division of Global Health Equity, Brigham and Women's Hospital, Boston, Massachusetts, United States of America; Iran University of Medical Sciences, Iran (Islamic Republic of)

## Abstract

**Background:**

American Indians and Alaska Native (AI/AN) populations experience significant health disparities compared to non-Hispanic white populations. Cardiovascular disease and related risk factors are increasingly recognized as growing indicators of global health disparities. However, comparative reports on disparities among this constellation of diseases for AI/AN populations have not been systematically reviewed.

**Objectives:**

We performed a literature review on the prevalence of diabetes, metabolic syndrome, dyslipidemia, obesity, hypertension, and cardiovascular disease; and associated morbidity and mortality among AI/AN.

**Data sources:**

A total of 203 articles were reviewed, of which 31 met study criteria for inclusion. Searches were performed on PUBMED, MEDLINE, the CDC MMWR, and the Indian Health Services.

**Study eligibility criteria:**

Published literature that were published within the last fifteen years and provided direct comparisons between AI/AN to non-AI/AN populations were included.

**Study appraisal and synthesis methods:**

We abstracted data on study design, data source, AI/AN population, comparison group, and. outcome measures. A descriptive synthesis of primary findings is included.

**Results:**

Rates of obesity, diabetes, cardiovascular disease, and metabolic syndrome are clearly higher for AI/AN populations. Hypertension and hyperlipidemia differences are more equivocal. Our analysis also revealed that there are likely regional and gender differences in the degree of disparities observed.

**Limitations:**

Studies using BRFSS telephone surveys administered in English may underestimate disparities. Many AI/AN do not have telephones and/or speak English. Regional variability makes national surveys difficult to interpret. Finally, studies using self-reported data may not be accurate.

**Conclusions and implications of key findings:**

Profound health disparities in cardiovascular diseases and associated risk factors for AI/AN populations persist, perhaps due to low socioeconomic status and access to quality healthcare. Successful programs will address social determinants and increase healthcare access. Community-based outreach to bring health services to the most vulnerable may also be very helpful in this effort.

**Systematic review registration number:**

N/A

## Introduction

It is recognized that American Indian and Alaskan Native (AI/AN) populations, defined as all people who originate from and maintain tribal affiliation or community attachment with any of the original inhabitants of North, South or Central America, are affected by health disparities compared with other populations in the United States. For instance, AI/AN have an average life expectancy of 5.2 years less than that of the general US population [1]. In fact, health statistics among AI/AN are sometimes closer to those found in lower- and middle-income countries, in part reflecting disparities in socioeconomic status, including worse living conditions, lower income, and greater barriers to health services compared with non-minority populations in the United States. The AHRQ National Healthcare Disparity annual report in 2009 found that AI/ANs received worse care than Whites for about 30% of quality measures and had worse access to care than Whites for 62% of access measures [2]. The report also found that disparities are not narrowing with improvements in health care. In fact, about six percent of quality measures had worsening disparities between 2002–2003 and 2007–2008 and 84% had stable disparities [2].

Among marginalized populations within the United States, AI/AN represent perhaps one of the populations most marked by health disparities. Prevalence of current smoking among youth (aged 12–17) and adults (over 18 years) and binge drinking is highest among AI/AN compared to other minority populations. AI/AN have similar rates of high school completeness, inadequate housing and poverty levels of African Americans, and higher than other minority groups [3].

Of growing global importance is the surge of non-communicable diseases (NCDs) as an indicator of health disparities. In the United States, NCDs are prevalent among minority populations and have generally been associated with health behaviors associated with lower income, such as lack of exercise and consumption of less healthy food. A WHO report found that NCDs account for 87% of all deaths in the United State and 63% of all deaths worldwide [4]. In particular, the contribution of cardiovascular disease (CVD) and associated risk factors – such as diabetes, hypertension, and dyslipidemia – to morbidity, mortality and healthcare costs are projected to rise in the coming decade, both in the U.S. and throughout the world [4].

There are about 6.2 million people classified as AI/AN as of 2011, comprising approximately 2.0% of the US population [5] living on 569 federally recognized tribes [6]. According to 2000 data, 43% live in the West, 31% live in the South, 17% live in the Midwest and 9% live in the Northeast [6]. While 34% of AI/AN reside on reservations or in rural areas, another 55% live in urban communities [7]. Because many national surveys have included data on race, there are numerous published reports which draw upon national statistics to provide statistics on health problems among AI/AN. It is generally recognized the cardiovascular health indices among AI/AN are worse than any other ethnic group in the United States. Nonetheless, to date, there has been no comprehensive review of the published literature on the burden of CVD and related risk factors among AI/AN compared with the rest of the U.S. population. To address this knowledge gap, we performed a systematic literature review on the prevalence of CVD and associated health problems among AI/AN compared with the rest of the U.S. population.

## Methods

One author (RH) conducted a systematic review of published literature reporting rates of diabetes, dyslipidemia, obesity, hypertension, and cardiovascular disease among AI/AN populations. We further limited our selection to cohort or randomized studies that permitted comparison of rates among AI/AN populations with non-AI/AN or general populations. Articles were included if they were published in the last fifteen years (1/1/1997–12/31/2012). Articles were excluded if discrete data were not reported for AI/AN racial status, or if they focused on children (below 18 years of age). We did not perform a systematic quality assessment of the studies, but rather sought to be inclusive of all studies, while raising critiques as part of the literature review. We made the decision to be all-inclusive because of the limited number of studies available for review.

Standard scientific databases including PubMed and MEDLINE were used. Keywords searched included combinations of the terms “Native American,” “American Indian,” “Alaska Native” and “health status,” “health disparities,” “diabetes,” “cardiovascular disease,” “stroke,” “acute MI,” “heart disease,” “metabolic syndrome,” “hypertension,” “obesity,” “hyperlipidemia.” All possible combinations of terms were searched. For example, for the obesity section, we performed two searches “obesity” and “American Indian” as well as “obesity” and “Alaska Native.” For cardiovascular diseases, we searched “cardiovascular disease” and “American Indian,” “stroke” and “American Indian,” “heart disease” and “American Indian,” and “myocardial infarction” and “American Indian.” We also did each of those terms combined with Alaskan Native. Early on, we explored whether or not there was a difference in searches performed using “American Indian” compared with “Native American” and we found no such difference. Searches were also performed on the Center for Disease Control and Prevention (CDC) and Morbidity and Mortality Weekly Report (MMWR) websites. Publications and reports prepared by the IHS were also accessed from their website. Additionally, reference lists from each relevant article were reviewed and cited articles were accessed if appropriate.

We assessed six health conditions, defined as follows. Obesity was defined as body mass index, i.e. the weight in kilograms divided by the square of the person's height in meters, of greater than 30 kg/m^2^. In most studies, this was based on self reported height and weight; a few of the studies used findings from physical exams or chart reviews. Metabolic syndrome was defined as the constellation of clinical findings that are associated with an increased risk of developing cardiovascular disease, as defined by National Cholesterol Education Program's Adult Treatment Panel III guidelines [8]. Diabetes, hypertension and high cholesterol were defined based on respondent affirmations, biometric measurements confirming the above conditions, chart reviews or analysis of Indian Health Service outpatient database. Cardiovascular disease was defined as the presence of coronary heart disease, angina, heart attack, stroke, or any other heart condition or disease, again based on self-reported conditions in surveys, analysis of death certificates, or review of tribal and Indian Health Service records.

## Results

### Summary of studies and data sources

A total of 203 articles were reviewed, of which 31 met criteria for inclusion. Of those, 56 total outcomes were addressed. (see **Figure S1**). Obesity was addressed in 12 studies,hyperlipidemia was addressed in 13 articles, diabetes in 18 studies, cardiovascular diseases, stroke and myocardial infarction was addressed in 11 articles, and metabolic syndrome in two studies. Studies are summarized in **Tables S1 and S2**.

Several large surveys provided data for numerous publications. Seven publications were based entirely on the national Behavior Risk Factor Surveillance System (BRFSS). Many other articles used data obtained from the BRFSS as a comparison group. The BRFSS study is an annual cross-sectional random-digit dialing telephone survey administered to adults 18 years or older and is used to track health conditions and risk behaviors [9].

The National Health Interview Survey (NHIS) database served as the data source for one publication and was also used to provide a comparison group for 10 additional studies where the data for AI/AN communities was gathered through other means. The NHIS was a cross-sectional household interview survey that has been ongoing since 1956. The study used a multistage area probability design sampling. Data were obtained about all household members, and more detailed information was collected from one sample child and one sample adult [10].

Two publications drew from data collected in the Racial and Ethnic Approaches to Community Health (REACH) project. This project was initiated in 2001 by the CDC to increase sampling among minority (including AI) populations. This survey used telephone interviews for some of the communities, including an AI community in Oklahoma, but also used face-to-face interviews where telephone coverage was believed to be less than 80% (including an AI community in North Carolina). The questionnaire was identical to the BRFSS questionnaire, facilitating comparisons to the US population. The REACH study was repeated in 2009 and included the same tribal participants above, as well as two additional AI groups, one in Michigan and a state-wide AI population in Oklahoma. Surveys were delivered either by telephone, mailing or face-to-face, and subjects were randomly selected by address, rather than telephone number in order to sample households without telephone service. Data were presented stratified by region and gender; however median values for each finding were reported in aggregate for AI populations surveyed across all regions.

The Strong Heart Study provided data for two of the publications reviewed. The main objective of the Strong Heart Study was to better understand cardiovascular disease and its risk factors among AI men and women. It recruited AI/AN aged 45 to 74 between 1989 and 1992 and included participants from 13 AI tribes in three geographic areas (Phoenix, Arizona, southwestern Oklahoma and central North/South Dakota). Four phases of the study were conducted, with a fifth currently underway [11].

### Obesity

Twelve articles compared rates of obesity among AI/AN communities versus other racial/ethnic groups. BRFSS survey data from 1997 through 2000 revealed higher rates of self-reported obesity for AI/AN respondents compared to respondents from all other racial/ethnic groups (23.9% versus 18.7%) [12]. Aggregated BRFSS data from a later cohort (2000 through 2006) suggest that obesity is disproportionately on the rise among AI/ANs compared with the non-Hispanic white participants (29.6% compared with 20.9%) [13]. Similarly NHIS data between 2004 and 2008 found even higher rates of obesity among all racial/ethnic groups, but in particular among AI/AN populations in whom 39.4% were obese compared with 24.3% among non-Hispanic white [6]. Higher rates of obesity are also observed among older AI/AN populations (>50 years of age) compared with non-Hispanic whites (29.2% vs 22.7%; p-value <0.05) based on BRFSS data between 2001 and 2004 [14].

There has also been a fair amount of literature comparing rates of obesity among AI/AN women with other populations. Using BRFSS data between 1998 and 2000, Doshi and Jiles found that AI/AN women had a higher rate of obesity compared with non-AI/AN women (26.8% compared with 19.3%) [15]. In the forementioned-study based on BRFSS data from 2000–2006, differences in obesity rates were slightly more pronounced for women (28.8% compared with 19.3%) compared with men (30.2% compared with 22.4%) [13]. Aggregated BRFSS data from 2001 and 2002 similarly showed that the prevalence of obesity in participants over the age of 55 was 50% higher among AI/AN women compared with non-Hispanic white women [16]. Amparo et al used BRFSS data from 2005 and 2007 to look at rates of obesity among AI/AN women of reproductive age and found that they were significantly more likely to report being obese than non-Hispanic women (25.8% versus 19.2%; p-value 0.001) [17]. Another national study (WISEWOMAN project from 2001 and 2002) screened low-income women (ages 40–64) who were participating in a National Breast and Cervical Cancer Early Detection Program; they found that the average BMI among AN was significantly higher compared with non-hispanic white participants (31.6 versus 29.2) [18].

In terms of regional data, obesity rates across regions and communities are consistently higher among AI/AN populations compared with non-AI/ANs; however the highest rates of obesity reported were among certain tribes of North Carolina. Harwell et al reported on BRFSS data, which was administered in 1999 to Indians living on or near Montana reservations and to non-Indian Montanans. He found that the prevalence of obesity was higher in the AI compared with the non-Indian population (38% vs 16%); the rate of being overweight was also much higher in the AI population (80% vs 54%) [19]. This difference was most pronounced among women, among whom the risk of obesity (odds ratio adjusted for age) was 3.57 times that for non-Indians (95% CI 2.35–5.41). Similarly, the 2001–2022 REACH study reported a much higher prevalence of obesity compared with state-specific BRFSS data. In Oklahoma, 32.6% of AI/ANs were obese compared to state-wide rates of 24.5% for men and 20.8% for women. North Carolina AI communities had extremely high rates of obesity among AI men and women (45.7% and 42.3%, respectively), much higher than the general NC populations (22.6% for men and 23.2% for women) [20]. The rate of obesity for these two AI communities was higher than any of the other minority groups surveyed for the study [20].

The second REACH study in 2009 revealed that obesity had increased in AI communities: 46.2% for men and 45.5% for women compared with national BRFSS median rates of 28.6% for men and 26.0% for women [21]. Regionally-stratified data revealed that obesity rates were highest in the Eastern Band of Cherokee Indians in North Carolina, where more than half of all individuals were obese, much higher than the rates reported for state-wide BRFSS participants (53.6% AI men vs 29.9% non-AI men; 50.2% AI women vs 29.8% non-AI women) [21].

Hodge et al performed a cross-sectional household survey of 457 AI adults in rural California between 2002 and 2003 and found that 11.6% of AI women were morbidly obese (BMI>40) and 37.3% were obese (BMI>30) [22]. The authors noted these rates to be markedly higher than national rates reported for black women (36.6%) and white women (20.3%), and also higher than national rates among AI women nationwide (29.4%) [22].

### Metabolic Syndrome

Two studies provided data on comparative rates of metabolic syndrome. Schumacher et al measured the prevalence of metabolic syndrome using a convenience sample of Alaska natives living in 26 villages as well as members of the Navajo Nation in Arizona and New Mexico. Sufficient data to determine presence of metabolic syndrome (including fasting blood sugar) was obtained for 3498 Alaskan natives and 4534 Navajos between 2004 and 2006 [23]. Because women were oversampled in the Alaska native population, data were presented stratified by gender. The authors found that 34.9% of AI/AN men and 40.0% of AI/AN women had metabolic syndrome, versus 24.8% and 22.8% among non-Hispanic men and women, respectively, based on NHANES data from 1988–1994 [23].

Another cross-sectional study measured the prevalence of metabolic syndrome between 2003 and 2006 among 4,457 AI individuals living on or near reservations in the Northern Plains and Southwestern US [24]. They found that the overall age-adjusted prevalence of the metabolic syndrome was 49.8% (95% CI: 47.8–50.7), compared with 34.0% in the general population based on NHANES data during the same period [24]. In sub-group analyses, they found that the prevalence of metabolic syndrome in non-diabetic AI men between the ages of 20–39 years was nearly twice the rate for similarly aged non-Hispanic whites without diabetes (39.2% compared to 20%) [24]. The differences in rates of metabolic syndrome were also striking among younger individuals, in which more than half of the AI population under 40 had metabolic syndrome compared with 20.3% of the younger NHANES population.

### Hypertension and Hyperlipidemia

Thirteen articles looked at rates of hypertension and hyperlipidemia among AI/AN populations. Three national studies using NHIS and BRFSS data evaluated rates of self-reported hypertension and hyperlipidemia with mixed results. One study based on 2003 BRFSS data showed higher rates of self-reported hypertension in AI/AN compared with non-Hispanic whites (26.8% versus 21.9%) [25]. Among AI/AN reporting hypertension, 61.3% reported taking antihypertensive medications compared with 60.9% of non-Hispanic white respondents [25]. 38.4% of AI/AN hypertensive respondents reported meeting physical activity recommendations compared with 42.9% of non-Hispanic whites [25].Using NHIS data between 2004 and 2008, the CDC reported increased rates of hypertension among AI/AN compared to non-Hispanic whites (34.5% vs 25.7%) [6]. In contrast, aggregated BRFSS data between 2001 and 2004 revealed no significant differences in hypertension or hypercholesterolemia among AI respondents compared to non-Hispanic whites (47.4% versus 44.2% for hypertension, 40.0% versus 42.5% for hypercholesterolemia) [14].

Data comparing rates of hypertension and hyperlipidemia among women were also equivocal. A study using BRFSS data from 2005–2007 found that significantly more AI/AN women (ages 18–44) reported having hypertension compared with non-Hispanic white women (12.0% vs 8.2%; p-value 0.007). No differences were found for those reporting a diagnosis of hyperlipidemia (19.7% of both groups reported this) [17]. The WISEWOMAN project (2001–2002) found AN women had a marginally lower average systolic blood pressure compared with non-Hispanic whites (120.5 mm Hg compared with 127.4 mm of Hg; furthermore, AN women had lower total cholesterol levels compared with non-Hispanic white women (209.3 v 217.2) [18]. Analysis of baseline data for participants in the Women's Health initiative study revealed similar rates of prehypertension in AI participants compared with white women (38.7% vs 39.5%) and higher rates of hypertension in AI participants compared with white women (40.6% vs 32.7%) [26].

In terms of regional data, Harwell et al analyzed 1999 BRFSS data on Indians versus non-Indians in Montana, stratified by age [19]. They found that both younger (>45 year-old) and older (>45 year-old) groups had higher rates of hypertension, yet lower rates of hyperlipidemia. The aOR for the younger group was 1.75 (95% CI 1.16–2.65) for hypertension and 1.42 (95% CI 1.08–1.87) for the older age group [19]. Similarly, the 2001–2002 REACH study found higher rates of hypertension for the two AI communities surveyed, compared with state-wide rates; these differences were more marked in OK compared with NC (OK: 35.8% v. 29.1% among men and 33.1% v. 28.0% among women; NC: 40.5% v. 25.4% among men and 40.4% v. 28.9% among women) [20]. Self-reported rates of hyperlipidemia were also higher in these two AI communities (OK: 44.3% vs 29.2% for men, 36.2% vs 30.0% for women; NC 31.9% vs 27.1% for men, 31.2% vs 30.5% for women) [20]. The second REACH in 2009 charted rising trends in hypertension among AI communities with median rates among AI men and women of 43.9% and 41.7%, respectively, compared with 29.8% and 27.8%, respectively, among national BRFSS respondents [21]. Schumacher's fore-mentioned study of AI/AN from Alaska and southwestern United States between 2004 and 2006 reported hypertension in 46.4% of AI/AN men and 36.2% of AI/AN women, compared with 37.2% and 27.8% among non-hispanic white men and women, respectively, from the NHANES study (albeit from a different time period, between 1988 and 1994) [23]. The EARTH study on AN residents (2004–2006) also reported a higher prevalence of hypertension (13% of men; 11% of women) compared with NHANES reported numbers (numbers not reported); although numbers were not provided, the authors also reported higher rates of dyslipidemia (low HDL, high LDL) among AN versus others [27].

Levin et al combined results from three different trials including AI communities (Chippewa and Menominee tribes in Minnesota and Wisconsin, the Catawba tribe located largely in South Carolina as well as the Lumbee tribe in North Carolina). He found higher rates of hypertension for all tribes compared with state-wide rates, although differences were only statistically significant in Minnesota/Wisconsin tribes (30.5% vs 24.3%) [28].

Another study looked at rates of hypertension in a randomly-selected diabetic population from the 1998 Indian Health Services (IHS) Diabetes Care and Outcomes audit. They stratified data by age and found that 47% of AI/AN younger than 45 had a blood pressure greater than 130/85; 66% of those older than 45 had blood pressure greater than 130/85 [29]. There was regional variation where the rate of hypertension varied from 37% in the Pacific area to 60% in the Great Lakes in the younger age group and 60% in the Pacific to 70% in Alaska in the older age group. The authors note that the rates in most of these populations were significantly higher than those reported for the NHANES III diabetic population where only 39% had hypertension [29].

### Diabetes

Eighteen studies reported data on diabetes. One combined BRFSS data from 1997 through 2000 and showed that the prevalence for AI/AN was self-reported to be 9.7% (95% CI 8.3, 11.1) compared with a prevalence of 5.7% (95% CI 5.6, 5.8) for all other races [12]. Analyses of later BRFSS surveys reveal a marked rise in diabetes among AI/AN: the prevalence of diabetes among AI/ANs in 2000–2006 had risen to 12.4%, whereas the rate among non-Hispanic whites remained relatively stable at 6.0% [13]. NHIS data between 2004 and 2008 corroborate this trend, revealing diabetes in 17.5% of AI/AN respondents compared to 6.6% of non-Hispanic whites (17.5% vs 6.6%) [6]. Rios Burrows et al also described this alarming rise in the rate of diabetes among AI/AN, using the IHS national outpatient database from 1990 through 1997. She found that the rate (both crude and age-adjusted) increased by 29% over this period [30]. This trend varied by region, ranging from a 16% increase in the Northern Plains to 76% in the Alaska region. The authors compared these rates of increased prevalence to national increases of 14% in the general U.S. population during this period [30]. The age-adjusted prevalence was found to be almost three times the prevalence among US non-Hispanic whites. A MMWR report also used the IHS national outpatient database to identify diabetes prevalence for each year between 1994 and 2002 and compared these rates to the prevalence of diabetes during the same time period based on BRFSS national results. The study revealed that the age-adjusted prevalence of diabetes increased by 33.2% (from 11.5% to 15.3%) for the AI/AN population while the prevalence of diabetes in all US adults rose by 54.0% (4.8% to 7.3%) [31]. Notably, the age-adjusted prevalence of diabetes in AI/AN adults was more than twice that of US adults for each year of the study [31].

This trend is further complicated by the growing number of young individuals affected by diabetes among AI/AN. In the previously-mentioned study, Rios Burrows et al also noted that AI diabetics were younger than the US general population diabetics (24% of AI diabetics were 65 or older compared with 45% of US diabetic population). Acton et al also used the IHS national outpatient database between 1990 and 1998 to investigate changes in prevalence of diabetes among children (<15 years), adolescents (15–19 years) and young adults (20–34 years) [32]. She found that the number of children, adolescents and young adults with diabetes increased by 71% from 4534 to 7736 persons. The prevalence increased by 47% for adults 20–24 years of age and by 50% for adults 25–34 years old. She compared these rates to a rise of 14% in the general US population under 45 years of age during the same time period [32].

Among older populations, BRFSS aggregated data from 2001 and 2002 revealed the prevalence of diabetes in the older population (>55 years) was 21.9% among AI/AN (95% CI 18.8, 24.9) compared with 13.0% of non-Hispanic whites (95% CI 12.6, 13.3), with an adjusted odds ratio was 1.66 (95% CI 1.37–2.00) [16]. Balluz et al found similar results when she combined BRFSS data from 2001 through 2004 for those older than 50 years of age: 22.9% of AI reported having diabetes compared to 12.0% of non-Hispanic white respondents (p-value <0.05) [14].

Among women, a recent study using BRFSS data from 2005 and 2007 found that 5.4% of AI/AN women of reproductive age (18–44 years-old) had diabetes, compared with 2.2% among non-Hispanic whites (p<0.001) [17]. Data from the WISEWOMAN study also found that AN women had a significantly higher rates of diabetes compared to non-Hispanic whites (10% vs 6%, p<0.05) [18].

As for regional data, Harwell analyzed 1999 BRFSS data administered in Montana in 1999, stratified by age [19]. The adjusted odds ratio for diabetes was 2.06 for AI/AN less than 45-years-old (95% CI .95–4.43) and 3.46 for AI/AN older than 45-years old (95% CI 2.35–5.09), compared with non-AI/AN state residents [19]. Notably, when stratified by gender and adjusted for age, his results revealed that AI women had more than three and-a-half times the rate of diabetes compared with non-AI/AN state residents (AOR 3.62; 95% CI 2.27–5.74) [19].

Both REACH studies assessed diabetes. In the first study, differences in rates of diabetes were especially substantial in the North Carolina population where 20.5% of AI men and 26.8% of AI women reported being diabetic compared to statewide rates of 6.8% for men and 6.7% for women [20]. The rates were also higher in Oklahoma where 11.9% of AI men reported being diabetic compared to 8.3% statewide and 12.2% of AI women compared to a statewide rate of 7.2% [20]. The second REACH study revealed a climbing rate of diabetes: a median of 18.0% and 18.4% of male and female AIs had diabetes, compared with national rates of 8.8% and 8.2%, respectively [21]. When stratified by region and gender, the rates were significantly increased for AI women in all four communities and for AI men in two out of four communities when compared to state-wide BRFSS data [21].

In the Phoenix area, O'Connell et al compared rates of diabetes in the AI population under the age of 65 receiving their care in Phoenix area IHS facilities (n = 30,121) with US insured adults (n = 1,500,002) matched to the AI population by age and sex in 2005. They found that prevalence of diabetes was 3.5 times higher for the AI population compared with the US population. When stratified by age, the prevalence was higher for every age group though the difference was most striking for the 35–44 year-olds where there was a 4-fold-increase in the prevalence of diabetes [33].

There are several studies that focused on one or several tribes. Will et al analyzed data on AI 20-years or older living on or near the Navajo reservation recruited between 1991 and 1992 in a random three-stage clustering design [34], 14.4% of Navajo adults reported a history of diabetes; of those without diabetes, an oral glucose tolerance test (OGTT) was administered. 6.8% of those tested were newly found to be diabetic based on the OGTT and 13.6% had impaired glucose tolerance. The age-adjusted total prevalence of diabetes was 22.9%. The authors compared this rate to the prevalence of the US general population in 1990 (5.2%) and concluded that the rate of diabetes in the Navajo nation was more than 4-times higher than the rate in the general US population [34]. Lee et al measured the prevalence of diabetes, based on fasting blood glucose, among 2205 randomly-selected enrolled members of the Cherokee Nation under 40 years of age [35]. They found that the overall age-adjusted prevalence of type 2 diabetes was 4.3%, compared to 1.1% within the U.S. general population in approximately the same period and age group based on NHANES III [35]. Levin et al's forementioned study also assessed the rate of diabetes among Chippewa, Menominee, Catawba and Lumbee tribes compared with state-wide BRFSS data. He found that the Chippewa and Menominee tribes had a nearly four-fold increase in the rate of diabetes (20.1% vs 5.8%) [28]. The Catawba tribe had more than a two-fold increase in the rate of diabetes (14.9% vs 6.6%), while the Lumbee tribe had an increased rate of diabetes that was not statistically significant (9.5% v. 6.8%) [28].

Several studies have looked at diabetes control and morbidity in AI/AN populations compared with US populations. Rith-Najarian et al analyzed the Indian Health Service Diabetes Care and Outcomes Audit to investigate regional variation in diabetes control. Among AI/ANs less than 45 years of age, 51% of patients had hemoglobin A1c greater than 9.0%; for those over the age of 45, 37% had a hemoglobin A1c greater than 9.0% [29]. Regional variation was marked. In Alaska, 27% of AN under age 45 had HbA1c greater than 9.0% whereas 56% of AI in the Southwest had a hemoglobin A1c greater than 9.0% [29]. In the older age group, the rates of poor control were similarly highest in the Southwest (42% of AI had A1c>9.0%) and lowest in Alaska (17% of AN had A1c>9.0%). The authors compared these rates of poor control with results from the diabetic population of the NHANES III study greater than 25 years, where only 25% of patients had HbA1c value greater than 9.0% [29].

In terms of morbidity, O'Connell et al reported that AI adults with diabetes in the Phoenix area were significantly more likely to be hypertensive (ratio of 1.9), have renal failure (ratio of 1.7), lower-extremity amputations (ratio of 14.4), neuropathy (ratio of 2.2), mental health disorders (ratio of 1.8), substance abuse (ratio of 14.9) and comorbid liver disease (ratio of 2.1) than were US adults with diabetes [33]. They derived a composite risk score of these comorbid conditions, which showed that AI adults with diabetes were 50% more likely to consume health resources than US adults with diabetes. They argued that in a commercial insurance environment, this difference would equate to an increase in yearly cost from $12,800 for the average US adult with diabetes to $19,260 for the average AI adult with diabetes per year [33].

### Cardiovascular disease

Eleven articles reported on cardiovascular disease morbidity and mortality. The CDC reported on results from the NHIS survey between 2004 and 2008 where the rate of self-reported heart disease, defined as presence of coronary heart disease, angina, heart attack or any other heart condition or disease, was higher among AI/AN respondents compared to Non-Hispanic whites (14.7% vs 12.2%) [6]. Similarly, the rate of stroke was higher amongst AI/AN compared to non-Hispanic whites (4.7% vs 2.4%) [6]. Data from the Strong Heart Study cohort also showed that cardiovascular mortality was higher for AI populations when compared with U.S. whites [36]. Data from the original Strong Heart Study cohort (1989–1992) were also used to analyze rates of fatal and non-fatal cardiovascular disease based on follow-up data for an average of 4.2 years [37]. They found that the incidence rate for fatal cardiovascular disease was 4.0 per 1000 for women (3.3 for fatal coronary heart disease and 0.8 for fatal stroke) and 9.1 per 1000 for men (8.0 for fatal coronary heart disease and 1.1 for fatal stroke). The rate for nonfatal cardiovascular disease was 7.8 per 1000 for women (6.1 for coronary heart disease and 2.0 for stroke) and 14.3 per 1000 for men (12.2 for coronary heart disease and 2.5 for stroke) [37]. They also compared rates of coronary heart disease to a cohort that was 25% black and found that rates for coronary heart disease were almost two times higher among AI (average follow-up for comparison cohort was 5.2 years) [37].

Two studies used national data obtained from death certificates to look at rates of stroke and/or cardiovascular disease based on race/ethnicity. Ayala et al looked at death certificates form the National Center for Health Statistics to see if there were differences in stroke types based on gender and race/ethnicity between 1995 and 1998. They found that age-standardized stroke death rate was lower for ischemic stroke in AI/AN women (49.2 vs 79.3 per 100,000) and in AI/AN men (47.6 vs 65.3 per 100,000) as well as for intracranial hemorrhage in AI/AN women (10.7 vs 12.8 per 100,000) and in AI/AN men (9.9 vs 13.6 per 100,000) [38]. Rates of subarachnoid hemorrhage were higher in AI/AN women (6.0 vs 4.5 per 100,000) but similar in AI/AN men (2.3 vs 2.9 per 100,000) [38]. Although these results suggest that rates of stroke might be similar or even decreased, the reality is that death certificates are known to underreport AI/AN race. The National Center for Health Statistics reported that death rates might be underreported by as much as 21% for AI/AN populations [38]. Another study looked at mortality from heart disease and stroke and attempted to correct for the underreporting of AI/AN race. Adjustment factors were determined by results from an IHS study where deaths were matched between IHS records and the National Death Index and then looked to see how many of them had AI/AN race reported on the death certificate. Misclassification rates varied widely across areas. Rhoades used these adjustment factors, which resulted in an increase in mortality from diseases of the heart by 18% and an increase in mortality from cerebrovascular disease by 11% compared with rates reported by the National Center for Health Statistics [39]. The mortality rate from diseases of the heart was highest among AI/AN after adjustment and the disparities between AI/AN and white death rate increased over time. The increase in disparity was largely because of declines in the death rate for whites whereas the death rate remained stable for AI/AN [39]. Disparities in mortality from cerebrovascular disease also widened due to increased death rate amongst AI/AN and decreased death rate for whites. During the final time period of the study, 1996 to 1998, the mortality rate for heart disease was 157.1 for AI/AN vs 125.9 per 100,000 for whites. The mortality rate for stroke was 29.5 per 100,000 for AI/AN vs 24.0 per 100,000 for whites [39].

Another study followed those Strong Heart Study participants without a stroke at the time of recruitment (between 1989 and 1992) and through the end of 2004 to determine the incidence rate of stroke as well as one-year post-stroke mortality. The author found that the incident rate of stroke in this cohort was 384 per 100,000 person-years for 45–54 year-olds, 727 per 100,000 person-years for 55–64 years, and 1002 per 100,000 person-years for 65–74 year-olds [40]. Compared to two cohorts (a non-Hispanic White population in Minnesota collected in 1985 and 1989, and the Framingham Heart Cohort), the Strong Heart cohort had much a higher incidence of stroke [40]. Furthermore, the 1-year mortality rate was 33.1% for women and 31% for men, compared with 24% and 21% respectively, based on pooled data from Framingham Heart Study, Atherosclerosis Risk in Communities Study and Cardiovascular Health Study. The author concluded that post-stroke mortality among AI was 1.5 times that of other US populations [40].

The above CDC report by Barnes et al also noted greater disparity in terms of heart disease among AI/AN women compared with non-AI/AN women (15.0 vs 11.3%) related to disparities among men (15.5 vs 13.5%) [6]. Similarly, the authors of the Strong Heart follow-up study compared incidence rates of stroke to a mostly white population and found that the rate for stroke was lower for AI men and similar for AI women, although the average follow-up for comparison cohort was 3.3 years compared to 4.2 years for the Strong Heart Study [37].

Regional data have borne out the national findings described above, with evidence of regional variation. In 1999, Harwell et al administered a telephone study using the BRFSS questionnaire to compare AI living on or near reservations in Montana with non-Indian Montana residents. When stratified by age, the rate of cardiovascular disease among AI was significantly higher with an aOR of 2.42 (95% CI 2.06–4.60) [19]. This difference was significant for both genders (men: aOR 2.15, 95% CI 1.28–3.61; women: aOR 2.25, 95% CI 1.29–3.93). In another study, also in Montana, Harwell et al compared age-adjusted heart disease and stroke mortality based on death certificates in that state between 1991 and 1995 and then between 1996 and 2000 [41]. He found that the death rate for heart disease was significantly higher for both time periods compared with white (for the later time period, the death rate was 328 per 100,000 for AI/AN vs 216 per 100,000 for whites). Similarly, he found that the rate of stroke-related mortality was also significantly higher for both time periods (for the later time period the rates were 81 per 100,000 for AI/AN vs 60 per 100,000 for white) [41]. Notably, and similar to the national data reported above, the disparity in stroke mortality rate increased over time largely due to the fact that stroke mortality improved only for white Montanans. 2001–2002 REACH data also revealed higher rates of cardiovascular disease (i.e. diagnosis of heart attack, angina, coronary heart disease or stroke) among AI compared with BRFSS data on the general population (men: 18% v. 9.5% in Oklahoma, 15.3% v. 9.1% in North Carolina; women: 12.9% v. 8.9% in Oklahoma, 13.2% v. 6.8% in North Carolina) [20]. 2009 REACH data confirmed similarly higher rates of cardiovascular disease among AI compared with national statistics (men 13.4% v. 8.8%; women 12.3% v. 6.3%) [21]. The Strong Heart Study mentioned above revealed greater disparities in the Dakotas, where mortality for AI aged 55–64 years old was 2.4 times that of whites [36]. A final study failed to observe significant differences in cardiovascular disease among three AI populations versus state populations: Levin et al found higher though not statistically significant increases in reported history of heart attack or stroke for the Catawba tribe (11.1 vs 6.7%). No real difference was noted for the Lumbee tribes and comparisons for the tribes in the ITHP cohort were limited as no BRFSS respondents in Minnesota and Wisconsin reported a history of stroke or heart attack [28].

## Discussion

This literature review highlights several salient findings. First, across a broad spectrum of chronic conditions, AI/ANs have disproportionately high rates of health problems and potentially higher rates of mortality from these conditions. Some of these disparities may be even greater among subgroups, such as women (obesity), or young adults (diabetes). Furthermore, reports drawing from serial BRFSS surveys also highlight disturbing trends in the rates of chronic conditions, including rising rates of self-reported obesity, and a stark rise in diabetes. Although survey methods may have varied over time, these trends suggest that AI/ANs have become increasingly vulnerable to such health risks over the past decade.

Despite the results summarized here, there is relatively scant literature that seeks to deepen our understanding of the causes underlying these health disparities. Furthermore, rates of disparities are largely based on large telephone-administered surveys such as BRFSS. As a result, exact rates and factors responsible for these disparities are not known. However, education, income and rates of unemployment have all been shown to be important factors in explaining disparities for other disadvantaged populations and are likely also key reasons for AI/AN health disparities. Nearly a quarter of AI/AN families are living below the federal poverty line, a rate that is 143% higher than non-Hispanic whites [42]. Similarly, 20.5% of AI/AN adults have not completed high school compared to a rate of 10% of non-Hispanic whites (both percentages are age-standardized) [42]. Unemployment rates have also been shown to be as much as three times higher in AI/AN populations [16]. All of these socioeconomic factors likely contribute to health disparities. In fact, some studies found that health differences in between AI/AN populations and non-Hispanic white populations were mitigated or sometimes eliminated, once they adjusted for factors such as economic status and education, suggesting that social determinants of health play a large part in the noted disparities [17].

Housing has also been shown to be linked to health outcomes, including increased rates of chronic and infectious diseases [43]. The American Housing Survey conducted by the Census Bureau found that that AI/AN population had nearly two times the rate of inadequate housing, defined as houses with moderate or severe physical problems such as a lack of running water [43]. Additionally, AI/AN had the highest odds of all disadvantaged populations of living in unhealthy housing (OR of 1.6 when compared with non-Hispanic whites). Unhealthy housing was defined as housing that has characteristics likely to negatively affect health including presence of pests, absence of smoke detector, water leaks, peeling paint in a house likely to have lead paint [43].

Food insecurity is also high among AI/ANs living on reservations [44]. The nutritional status of AI/ANs has dramatically shifted in the past few decades from one of under-nutrition to that of poor nutrition characterized by excessive caloric intake of food with poor nutritional content [45], [46]. Access to healthy foods, especially fresh fruits and vegetables, are impeded by low income, geographical isolation, and the paucity of inexpensive vendors on reservation land, resulting in food deserts for many AI/AN nations [47], [48], [49]. Sustained, wide-scale, traditional agriculture has been challenged by limited water access, pest control, and regulatory requirements for commercial production [50]. Recently, there has also been a proliferation of fast-food restaurants near AI/AN communities, which is likely linked to increased consumption of unhealthy foods [51].

Despite the existence of the IHS, which theoretically provides universal health services to AI/AN, there is a surprising number of AI/AN who report being uninsured. In fact, AI/AN are more likely than non-Hispanic whites to report no usual source of healthcare or health insurance, particularly those who live off of reservation land [13]. Despite the fact that 60% of AI/AN currently live outside of their home reservations, only 1% of the IHS budget is used for AI/AN healthcare off of the reservations [52]. Even for those who receive care through Indian Health Services, the per capita funding for IHS is less than half of what is provided to Medicaid and/or incarcerated populations [52]. The amount of money allocated to each IHS patient is just over one third of what is allocated to the general population overall (1351 compared with 3766) [53]. Cultural differences between the mostly non-AI/AN providers in the IHS and AI/AN patients may contribute to distrust in doctors and the medical system, which has been implicated as a possible cause for poorer health outcomes in other disadvantaged populations [54].

In order to effectively narrow the health disparities described in this review, structural changes must occur to address the root causes of disparities. This includes addressing poverty, gaps in education and employment opportunities as well as improving housing conditions and access to healthy foods. Improved funding of the IHS generally and culturally-relevant programs, including community-based outreach to reach those who do not have access to facility-based services, is critical in order to narrow these disparities.

The community health representative (CHR) program and public health nursing (PHN) program could be especially effective in encouraging lifestyle and behavioral changes as well as facilitating access to health services. Notably, a recent AHRQ systematic review found that use of community health workers can improve health outcomes in underserved populations, supporting this conclusion [55]. The American Indian population is especially well-suited to such an intervention given the existence of the CHR program, which is already in place nationally in AI communities.

Our review had several limitations. Our review may not have been exhaustive because we did not include major databases such as ISI Web of Knowledge and Scopus. Our findings were also was limited by the inherent limitations of the literature itself. Many of the studies used BRFSS as the source for data. BRFSS telephone surveys are very likely to miss significant numbers of native people living on the reservations, where as many as 25% lack telephones [13]. This may result in an over representation of AI/AN living off of reservations and in urban locations. Such a sampling bias could result in an underestimate of disparities, since studies have suggested that AI/ANs living in households without telephones have higher rates of unhealthy lifestyles including tobacco use, infrequent physical activity and/or binge drinking [12]. Language is another barrier to participation in these surveys, as very few surveys were administered in native languages. Neither BRFSS nor NHIS surveys are translated into native languages. There is also significant evidence of regional variability, making national studies difficult to interpret. Most of the studies depended on self-reported knowledge of diseases. The accuracy of self-reported knowledge of diseases for AI/AN populations is not clear and has not been studied. Finally, cultural differences may impact the ways in which certain questions are answered.

In summary, our review synthesizes a robust body of literature that highlights the extent of NCD health disparities among AI/AN. These findings beg the urgency for further research aimed at understanding the linkage between these disease states to underlying structural causes and, in turn, to addressing and overcoming these health disparities.

## Supporting Information

Figure S1 **Flowchart illustrating citation selection process.**
(TIF)

Checklist S1 **PRISMA checklist.**
(DOC)

Table S1 **Summary of citations reviewed.**
(DOCX)

Table S2 **Outcomes of each study.**
(DOCX)
