# Supplementary material for: Systematic Review of Health Disparities for Cardiovascular Diseases and Associated Factors among American Indian and Alaska Native Populations
Source: PLoS One. 2014 Jan 15;9(1):e80973. doi: 10.1371/journal.pone.0080973 (PMC3893081; doi:10.1371/journal.pone.0080973)
Supplement: Table S1 — Summary of citations reviewed. (DOCX) [file pone.0080973.s003.docx]

Table S1. Summary of citations reviewed

| Author, year | AI/AN population | Comparison Group | Outcome | Comments |
| --- | --- | --- | --- | --- |
| Lee 1997 [36] | Tribes in the Strong Heart Study (see text), 1984-1988 | National non-Hispanic Whites, 1986 | Cardiovascular Disease Mortality (per 10,000) | Greater reliability of AI mortality estimates through use of multiple databases. Questionable comparison of regional AI population versus national control |
| Howard 1999 [37] | Strong Heart Study, 1989 -1995 | Stroke: CHS study (predominantly white cohort). CHD: ARIC study (25% black) | Fatal coronary heart disease (per 1000 person-years). Fatal stroke (per 1000 person-years). Nonfatal coronary heart disease (per 1000 person-years). Nonfatal stroke (per 1000 person-years) | Prospective cohort study. Exact numbers for comparison not provided. Comparison groups had different lengths of average follow-up and different populations. |
| Zhang 2008 [40] | Strong Heart Study cohort without stroke at baseline, 1989-2004 | Stroke incidence: White population in Minnesota (1985-1989) & Framingham Heart Study (FHS, 1980-2003). Mortality: Pooled data from FHS, atherosclerosis risk in communities study and cardiovascular health study. | Incidence rate of Stroke (per 100,000 person years), 30-day mortality, 1-year mortality | Observational cohort study. Comparison groups had different follow-up time periods and from very different regions of country |
| Levin 2002[28] | Red Lake Band, White Earth Band, Menominee (ITHP); Catawba tribe in North and South Carolina; Lumbee tribe east of the Mississippi. | Respective state wide BRFSS data | History of heart attack or stroke, Hypertension, Diabetes | Stronger comparison by matching controls by state and time period. Data collection method differed among studies (e.g. in-person v. telephone |
| Redwood 2010[27] | AN adults, 2004 & 2006 | NHANES data for all races, 1994-2004. | Obesity, Hypertension | Comparison limited by national rather (vs. regional AI sample) and self-reported data (vs. direct measurements for AI). Exact rates of obesity & hypertension for controls not reported, but “significantly higher” in all groups |
| Finkelstein 2004 [18] | Alaska Native females 40-64 years of age, WISEWOMAN 2001-2002 | White non-Hispanic women 40-64 years old in 8 locations in U.S., WISEWOMAN, 2001-2002 | Average body mass index, Average systolic blood pressure, Average total cholesterol, Diabetes | Study selected low-income women, thus less chance of confounding by socio-economic status |
| Hodge 2011[22] | AI women in California, 2002-2003 | Black & non-Hispanic white women, NHANES 2005-2006 | Obesity | Weaker comparison due to different time period and national population |
| Schumaker 2008 [23] | Alaska natives & Navajo, 2004-2006 | Non-Hispanic whites, NHANES 1988-1994. | Metabolic Syndrome | Weaker comparison due to different time period & national sample. Outcomes based on clinical measurements for AI/AN vs. self-report for comparison |
| Sinclair 2011[24] | AI/AN living in two Northern Plains and one Southwest community, 2003-2006 | Non-Hispanic whites, NHANES 2003-2006 | Metabolic Syndrome | Weaker comparison due to national sample. Outcomes based on clinical measurements for AI/AN vs. self-report for comparison |
| Amparo[17] | AI/AN non-pregnant women, 18-44 years old, BRFSS 2005-2007 | Non-Hispanic white non-pregnant women 18-44 years old, BRFSS 2005-2007 | Obesity, Hypertension, Hyperlipidemia, Diabetes | Comparable comparison group from same national survey |
| CDC 2003[12] | AI/AN, BRFSS 1997-2000 | All non-AI/AN, BRFSS 1997-2000 | Obesity, Diabetes | Comparable comparison group from same national survey |
| Denny[16] | AI/AN, BRFSS 2001 -2002 | Non-Hispanic Whites, BRFSS 2001-2002 | Obesity, Diabetes | Odds ratios adjusted for multiple socioeconomic characteristics. |
| Steele [[13](#_ENREF_13)] | AI/AN, BRFSS 2000-2006 | Non-Hispanic Whites, BRFSS 2000-2006 | Diabetes, Obesity | Results adjusted only for age |
| Balluz[[14](#_ENREF_14)] | AI/AN ≥ 50-years-old, BRFSS 2001-2004 | Non-Hispanic Whites ≥ 50-years-old, BRFSS 2001-2004 | Obesity, Diabetes, Hypertension, Hyperlipidemia | Results adjusted only for age |
| Barnes[6] | AI/AN, NHIS 2004-2008 | Non-Hispanic Whites, NHIS 2004-2008 | Heart disease, Stroke, Obesity, Hypertension, Diabetes | Face-to-face survey administered in the household. Results adjusted only for age. |
| Harwell[19] | AI on or near seven reservations in Montana, 1999 | Non-AI residents of Montana, 1999 | Cardiovascular Disease, Obesity, Hypertension, Hyperlipidemia, Diabetes | Comparison groups not well matched in age, hence age stratified analysis. Recruitment methods used to increase AI participation |
| Rith-Najarian[29] | AI/AN diabetics | NHANES III diabetic population | Hypertension, Diabetes control (% A1c > 9.0%) | Data collection methods differed (AI/AN chart review vs. NHANES self-report). Comparison group did not have same age-stratification. |
| Zhao 2008[25] | AI/AN women, BRFSS 2003 | Non-Hispanic White women, BRFSS 2003 | Hypertension, Hypertensive and taking medication, Hypertensive and physically active | Comparable comparison group from same national survey |
| Burrows 2000[30] | AI/AN in the IHS national outpatient database, 1990-1997 | General US population, BRFSS 1990-1996. | Increase in diabetes prevalence | Data collection methods differed (AI/AN chart review vs. BRFSS self-report). Results adjusted only for age. |
| Acton 2002[32] | AI/AN up to 34 years old in IHS national outpatient database, 1990 and 1998 | General US population up to 45 years-old, BRFSS, 1990 and 1996 | Diabetes | Data collection methods differed (AI/AN chart review vs. BRFSS self-report). Weaker comparison due to different age group and different years |
| CDC 2003 [31] | AI/AN in IHS outpatient database, 1994-2002 | U.S. respondents, BRFSS 1994-2002. | Diabetes | Data collection methods differed (AI/AN chart review vs. BRFSS self-report) |
| Will 1997[34] | Navajo ≥ 20 years old, 1991-1992 | General U.S. population, CDC data 1993 | Diabetes | Navajo cohort assessed with self-report and oral glucose tolerance test. Rate of diabetes estimated in comparison group as double self-reported rate. |
| Lee 2004[35] | Cherokee <40 years old, year? | National respondents 20-40 years old, NHANES 1988-1994 | Diabetes | Data collection differed (fasting blood sugar versus self-report). Weaker comparison group due to national sample, age range not completely matched |
| O’Connell[33] | Diabetic AI seen at Phoenix IHS, between 10/04 and 11/05 | U.S. insured adults matched by age and sex from 2005 MarketScan Research Database | Diabetes | Cohorts well-matched. Results not adjusted for socioeconomic differences (and comparison cohort was insured US adults) |
| Hsia[26] | AI in Women’s Health Initiative Study, 1993-1998 | Non-AI in same study | Hypertension | AI/AN only 1.6% of study cohort |
| Ayala[38] | AI based on national review of death certificates | Non-AI based on national review of death certificates | Ischemic Stroke, per 100,000. Intracranial hemorrhage, per 100,000. Subarachnoid hemorrhage, per 100,000 | AI/AN race underreported in death certificates. Cause of death on certificates not always accurate |
| Rhoades[39] | AI based on national review of death certificates | Non-AI based on national review of death certificates | Cardiovascular Disease, per 100,000. Cerebrovascular disease, per 100,000 | Results corrected for likely under-reporting of AI/AN race on death certificates. Cause of death on certificates not always accurate. |
| Harwell[41] | AI based on death certificate review in Montana, 1991 -2000 | Non-AI based on death certificate review in Montana, 1991 -2000 | Cardiovascular Disease, per 100,000. Stroke, per 100,000 | AI/AN race underreported in death certificates. Cause of death on certificates not always accurate |
| Doshi [15] | AI/AN women, BRFSS 1998-2000 | Non-AI/AN women, BRFSS 1998-2000 | Obesity | Results adjusted only for age |
| CDC 2004[20] | AI in Oklahoma (OK) & North Carolina (NC), REACH 2001-2002 | General population in OK & NC, BRFSS 2000-2001 | Obesity, Cardiovascular Diseases (history of myocardial infarction, angina, coronary heart disease or stroke), Hypertension, Hyperlipidemia, Diabetes | BRFSS survey used for REACH, thus comparable. However years used for comparison group slightly different than those used for REACH group. |
| CDC 2011[21] | Eastern Band of Cherokee Indians (NC), Choktow Nation (CN), Oklahoma (OK), Intertribal council of Michigan (MI), REACH 2009 | General population in OK, NC, OK & MI, BRFSS 2007-2009 | Obesity, Cardiovascular disease (history of myocardial infarction, angina, coronary heart disease or stroke), Hypertension, Diabetes | BRFSS survey used for REACH, thus comparable. However years used for comparison group slightly different than those used for REACH group. |

Abbreviations: AI: American Indian, AN: Alaska Native, ARIC: Atherosclerosis Risk in Communities, BRFSS: Behavior Risk Factor Surveillance System, CDC: Center for Disease Prevention and Control, CHS: Cardiovascular Health Study, FHS: Framingham Heart Study, IHS: Indian Health Services, ITHP: Inter-Tribal Heart Project, NHANES: National Health Interview Survey, U.S.: United States
