# Supplementary material for: Systematic Review of Health Disparities for Cardiovascular Diseases and Associated Factors among American Indian and Alaska Native Populations
Source: PLoS One. 2014 Jan 15;9(1):e80973. doi: 10.1371/journal.pone.0080973 (PMC3893081; doi:10.1371/journal.pone.0080973)
Supplement: Table S2 — Outcomes of each study. (DOCX) [file pone.0080973.s004.docx]

Table S2. Outcomes of each study

| Author, year | Outcome | Main findings (AI/AN versus comparison) |
| --- | --- | --- |
| Lee 1997[36] | Cardiovascular Disease Mortality (per 10,000) | For AZ: 45-54 yo: 21.4 vs 17.0, 55-64 yo: 69.4 vs 49.8; 65-74 yo: 109.8 vs 127.3. For OK: 45-54 yo: 17.0 vs 17.0; 55-64 yo: 54.4 vs 49.8; 65-74 yo: 141.4 vs 127.3. For N/SD: 45-54 yo: 35.4 vs 17.0; 55-64 yo: 117.4 vs 49.8; 65-74 yo: 167.0 vs 127.3 |
| Howard 1999[37] | Fatal coronary heart disease (per 1000 person-years) | For AZ: M: 19, W: 18. For OK: M: 22, W: 11. For N/SD: M: 26, W: 15 |
| Howard 1999[37] | Fatal stroke (per 1000 person-years) | For AZ: M: 4, W: 5. For OK: M: 2, W: 1. For N/SD: M: 3, W: 4 |
| Howard 1999[37] | Nonfatal coronary heart disease (per 1000 person-years) | For AZ: M: 24, W: 29. For OK: M: 21, W: 32. For N/SD: M: 30, W: 43 |
| Howard 1999[37] | Nonfatal stroke (per 1000 person-years) | For AZ: M: 5, W: 8. For OK: M: 6, W: 7. For N/SD: M: 10, W: 12 |
| Zhang 2008 [40] | Incidence rate of Stroke (per 100,000 person years) | SHS: for 45-54: 384. For 55-64: 727, for 65-74: 1002. MN study: for 45-54: 63, for 55-64: 273, for 65-74: 669. FHS: for 45-54: 320, for 55-64: 637, for 65-74: 972. |
| Zhang 2008 [40] | 30-day mortality | SHS: 18%, F: 18.5% |
| Zhang 2008 [40] | 1-year mortality | SHS: M: 31%, F: 33.1%. FHS: M: 21%, F: 24%. |
| Levin 2002[28] | History of heart attack or stroke | ITHP: 20.1% vs N/A, Catawba: 11.1% vs 6.7%, Lumbee: 7.8% vs 7.8% |
| Levin 2002[28] | Hypertension | ITHP: 30.5% vs 24.3%; p<0.05, Catawba: 33.9% vs 27.5%, Lumbee: 28.1% vs 25.9% |
| Levin 2002[28] | Diabetes | ITHP: 20.1% vs 5.8%; p<0.05, Catawba: 14.9% vs 6.6%; p<0.05 , Lumbee: 9.5% vs 6.8% |
| Redwood 2010[27] | Obesity | M: 68%, F: 78% |
| Redwood 2010[27] | Hypertension | M: 13%, F: 11% |
| Finkelstein 2004 [18] | Average body mass index | 31.6 vs 29.2; p<0.05 |
| Finkelstein 2004 [18] | Average systolic blood pressure | 120.5 vs 127.4 mm Hg; p<0.05 |
| Finkelstein 2004 [18] | Average total cholesterol | 209.3 mg/dl vs 217.2 mg/dl; p<0.05 |
| Finkelstein 2004 [18] | Diabetes | 10% vs 6%; p<0.05 |
| Hodge 2011[22] | Obesity | AI: 48.9%, Black: 36.6%, White: 20.3 |
| Schumaker 2008 [23] | Metabolic Syndrome | M: 34.9% vs 24.8%, F: 40.0% vs 22.8% |
| Sinclair 2011[24] | Metabolic Syndrome | 49.8% vs 34.0% |
| Amparo[17] | Obesity | 25.8% vs 19.2%; p-value 0.001 |
| Amparo[17] | Hypertension | 12.0% vs 8.2%; p-value 0.007 |
| Amparo[17] | Hyperlipidemia | 19.7% vs 19.7%; p-value 0.76 |
| Amparo[17] | Diabetes | 5.4% vs 2.2%; p-value <0.001 |
| CDC 2003[12] | Obesity | 23.9% (21.9-25.9) vs 18.7% (18.5-18.9) |
| CDC 2003[12] | Diabetes | 9.7% (8.3-11.1) vs 5.7% (5.6-5.8) |
| Denny[16] | Obesity | 29.3% vs 21.7%, aOR 1.29 (1.07-1.55) |
| Denny[16] | Diabetes | 21.9% vs 13.0%, aOR 1.66 (1.37-2.00) |
| Steele [[13](#_ENREF_13)] | Diabetes | 12.4% (11.4-13.4) vs 6.0% (5.9-6.1) |
| Steele [[13](#_ENREF_13)] | Obesity | 29.6% (28.3-30.9) vs 20.9 (20.7-21.0) |
| Balluz[[14](#_ENREF_14)] | Obesity | 29.2% vs 22.7%; p-value<0.05 |
| Balluz[[14](#_ENREF_14)] | Diabetes | 22.9% vs 12.0%; p-value<0.05 |
| Balluz[[14](#_ENREF_14)] | Hypertension | 47.4% vs 44.2% |
| Balluz[[14](#_ENREF_14)] | Hyperlipidemia | 40.0% vs 42.5% |
| Barnes[6] | Heart disease | 14.7% vs 12.2% |
| Barnes[6] | Stroke | 4.7% vs 2.4% |
| Barnes[6] | Obesity | 39.4% vs 24.3% |
| Barnes[6] | Hypertension | 34.5% vs 25.7% |
| Barnes[6] | Diabetes | 17.5% vs 6.6% |
| Harwell[19] | Cardiovascular Disease | <45 yrs: 9% vs 6%; aOR 1.01 (95% CI 0.36-2.88), ≥45 yrs: 71% vs 53%; aOR 2.42 (95% CI 2.06-4.60) |
| Harwell[19] | Obesity | <45 yrs: 29% vs 12%; aOR 2.99 (95% CI 2.08-4.30), ≥45 yrs: 38% vs 16%; aOR 3.08 (95% CI 2.24-4.24) |
| Harwell[19] | Hypertension | <45 yrs: 15% vs 10%; aOR 1.75 (95% CI 1.16-2.65), ≥45 yrs: 42 vs 36%; aOR 1.42 (95% CI 1.08-1.87) |
| Harwell[19] | Hyperlipidemia | <45 yrs: 8% vs 10%; aOR 0.83 (95% CI 0.53-1.32), ≥45 yrs: 24% vs 32%; aOR 0.68 (95% CI 0.51-0.92) |
| Harwell[19] | Diabetes | <45 yrs: 5% vs 2%; aOR 2.06 (95% CI 0.95-4.43), ≥45 yrs: 24% vs 9%; aOR 3.46 (95% CI 2.35-5.09) |
| Rith-Najarian[29] | Hypertension | <45 yrs: 47% vs 22%, ≥45 yrs: 66% vs 22% |
| Rith-Najarian[29] | Diabetes control (% A1c > 9.0%) | <45 yrs: 51% vs 25%, ≥45 yrs: 37% vs 25% |
| Zhao 2008[25] | Hypertension | 26.8% vs 21.9% |
| Zhao 2008[25] | Hypertensive and taking medication | 61.3% vs 60.9% |
| Zhao 2008[25] | Hypertensive and physically active | 38.4% vs 42.9% |
| Burrows 2000[30] | Increase in diabetes prevalence | 29% vs 14% |
| Acton 2002[32] | Diabetes | 20-24 yo: 47% vs 14%, 25-34 yo: 50% vs 14% |
| CDC 2003 [31] | Diabetes | 1994: 11.5% vs 4.8%, 2002: 15.3% vs 7.3% |
| Will 1997[34] | Diabetes | 22.9% vs 5.2% |
| Lee 2004[35] | Diabetes | 20-29 yo: 4.4% vs 1.1%, 30-40 yo: 9.2% vs 1.1% |
| O’Connell[33] | Diabetes | 18-34 yo: 3.8% vs 1.1%; p<0.05, 35-44yo: 12.2% vs 3.0%; p<0.05, 45-54yo: 22.9% vs 6.1%; p<0.05, 55-64yo: 33.5% vs 12.2%; p<0.05 |
| Hsia[26] | Hypertension | 40.6% vs 32.7% |
| Ayala[38] | Ischemic Stroke, per 100,000 | F: 49.2 vs 79.3 , M: 47.6 vs 65.3 |
| Ayala[38] | Intracranial hemorrhage, per 100,000 | F: 10.7 vs 12.8 , M: 9.9 vs 13.6 |
| Ayala[38] | Subarachnoid hemorrhage, per 100,000 | F: 6.0 vs 4.5 , M: 2.3 vs 2.9 |
| Rhoades[39] | Cardiovascular Disease, per 100,000 | 1992-1994: 157.6 vs 139.9 , 1994-1996: 156.0 vs 133.1 , 1996-1998: 157.1 vs 125.9 |
| Rhoades[39] | Cerebrovascular disease, per 100,000 | 1992-1994: 27.8 vs 24.5 , 1994-1996: 30.5 vs 24.7 , 1996-1998: 29.5 vs 24.0 |
| Harwell[41] | Cardiovascular Disease, per 100,000 | 1991-1995: 307 vs 237 , 1996-2000: 328 vs 216 |
| Harwell[41] | Stroke, per 100,000 | 1991-1995: 80 vs 64, 1996-2000: 81 vs 60 |
| Doshi [15] | Obesity | 26.8 ± 2.8 % vs 19.3 ± 0.3 % |
| CDC 2004[20] | Obesity | OK: M: 32.6% (27.0-38.8) vs 24.5% (22.0-27.0), W: 32.6% (27.7-37.9) vs 20.8% (18.8-22.8). NC: M: 45.7% (41.4-50.1) vs 22.6% (20.1-25.1), W: 42.3% (38.6-46.2) vs 23.2% (21.0-25.4) |
| CDC 2004[20] | Cardiovascular Diseases (history of myocardial infarction, angina, coronary heart disease or stroke) | OK: M: 18.0% (13.5-23.6) vs 9.9% (8.3-11.5), W: 12.9% (9.9-16.6) vs 8.9% (7.7-10.2). NC: M: 15.3% (12.3-18.8) vs 9.1% (7.1-11.0), W: 13.2% (10.9-15.8) vs 6.8% (5.5-8.1) |
| CDC 2004[20] | Hypertension | OK: M: 35.8% (29.7- 42.4) vs 29.1% (26.5-31.6), W: 33.1% (28.5-38.0) vs 28.0% (25.8-30.1). NC: M: 40.5% (36.2-45.0) vs 24.4% (22.8-27.9), W: 40.4% (36.9-44.1) vs 28.9% (26.7-31.0) |
| CDC 2004[20] | Hyperlipidemia | OK: M: 44.3% (36.8-52.0) vs 29.2% (26.0-32.3), W: 36.2% (30.7-42.2) vs 30.0% (27.6-32.3). NC: M: 31.9% (27.1-37.1) vs 27.1% (24.1-30.0), W: 31.2% (27.5-35.1) vs 30.5% (27.9-33.0) |
| CDC 2004[20] | Diabetes | OK: M: 11.9% (8.5-16.5) vs 8.3% (6.7-9.8), W: 12.2% (9.3-15.9) vs 7.2% (6.0-8.3). NC: M: 20.5% (17.2-24.2) vs 6.8% (5.4-8.1), W: 26.8% (23.6-30.3) vs 6.7% (5.7-7.6). |
| CDC 2011[21] | Obesity | NC: M: 53.6% (43.1-63.9) vs 29.9% (30.8-34.1), W: 50.2% (41.5-58.9) vs 29.8% (28.6-31.0). CN: M: 39.4% (33.8-45.2) vs 32.5% (30.8-34.1), W: 35.1% (31.0-39.4) vs 30.6% (29.4-31.7). MI: M: 43.1% (35.0-51.7) vs 30.3% (28.9-31.8), W: 55.1% (48.0-62.0) vs 29.5% (28.4-30.7). OK: M: 49.3% (42.8-55.8) vs 32.5% (30.8-34.1), W: 40.7% (35.9-45.8) vs 30.6% (29.4-31.7) |
| CDC 2011[21] | Cardiovascular disease (history of myocardial infarction, angina, coronary heart disease or stroke) | NC: M: 12.8% (7.4-21.0) vs 9.8% (9.0-10.8), W: 11.8% (7.7-17.5) vs 7.4% (6.9-8.0). CN: M: 16.5% (13.1-20.5) vs 11.3% (10.5-12.2), W: 13.5% (11.2-16.1) vs 9.2% (8.6-9.8). MI: M: 12.7% (8.4-18.6) vs 9.5% (8.8-10.2), W: 12.7% (8.7-18.3) vs 7.8% (7.3-8.3). OK: M: 14.0% (10.8-18.0) vs 11.3% (10.5-12.2), W: 11.1% (8.8-13.8) vs 9.2% (8.6-9.8). |
| CDC 2011[21] | Hypertension | NC: M: 45.9% (35.4-56.7) vs 31.7% (29.6-33.8), W: 41.4% (32.9-50.4) vs 31.4% (29.8-33.1). CN: M: 42.5% (37.1-48.2) vs 34.9% (32.7-37.1), W: 42.5% (38.5-46.7) vs 33.8% (32.3-35.4). MI: M: 33.7% (26.5-41.8) vs 31.8% (29.9-33.9), W: 36.4% (30.2-43.1) vs 27.8% (26.5-29.2) . OK: M: 45.2% (38.8-51.8) vs 34.9% (32.7-37.1), W: 42.0% (37.2-47.0) vs 33.8% (32.3-35.4) |
| CDC 2011[21] | Diabetes | NC: M: 30.4% (21.9-40.5) vs 9.2% (8.5-9.9), W: 23.4% (17.1-31.1) vs 9.7 (9.1-10.4). CN: M: 11.6% (8.9-15.1) vs 11.1% (10.2-12.0), W: 16.2% (13.6-19.2%) vs 10.1% (9.4-10.7). MI: M: 23.1% (16.8-30.9) vs 10.0% (9.3-10.8), W: 20.6% (15.5-26.8) vs 8.5% (7.9-9.0). OK: M: 12.9% (9.7-17.0) vs 11.1% (10.2-12.0), W: 15.4% (12.5-18.7) vs 10.1% (9.4-10.7). |
